# Health care worker experiences with a brief peer support and well-being intervention during the COVID-19 pandemic

**DOI:** 10.1186/s12913-025-13268-6

**Published:** 2025-09-30

**Authors:** George Timmins, Stephanie Williamson, Andrea Cassells, Katie Davis, Lu Dong, Jonathan N. Tobin, Courtney Gidengil, Lisa S. Meredith, Peggy G. Chen

**Affiliations:** 1 RAND, Santa Monica, USA; 2 Clinical Directors Network, New York, USA; 3 Vizient, Inc, Irving, USA; 4 Rockefeller University, New York, USA; 5 RAND, Boston, USA

**Keywords:** Healthcare workforce, Mental health, Stress first aid, COVID-19 pandemic

## Abstract

**Importance:**

Health care workers (HCW) faced chronic stress during the COVID-19 pandemic and were at high risk of illness, death and burnout.

**Objective:**

To understand the experiences of and assess the acceptability and usability of the “Stress First Aid” (SFA) intervention for HCWs.

**Design:**

We used a mixed methods approach to conduct: (1) a quantitative post-intervention survey of experiences with the SFA intervention within a cluster randomized controlled trial (cRCT); and (2) a qualitative descriptive analysis. The intervention was rolled out over three waves from March 2021 – October 2022 simultaneously with the sites’ COVID-19 response.

**Setting:**

Our team engaged and recruited eight pairs of hospitals and six pairs of Federally Qualified Health Centers (FQHCs), balanced across region, including nine states, and matched on size, type, and COVID-19 burden.

**Participants:**

A total of 862 HCWs received the SFA intervention and completed both the pre- and post-intervention surveys (FQHC *n* = 245 and hospital *n* = 617). For the qualitative analysis, among HCWs who agreed to be contacted for a post-intervention interview, we purposively sampled a subset of 35 HCWs balanced by site, gender, age, race/ethnicity and HCW type.

**Intervention:**

SFA is an evidence-informed intervention adapted to mitigate the psychosocial impact of COVID-19 on HCWs through individual peer support actions.

**Main Outcome(s) and Measure(s):**

Quantitative measures are binary indicators of agreement with 6 questions about experiences with the SFA intervention. For the qualitative analysis, we utilized a semi-structured interview protocol to provide additional context on experience with SFA and how SFA affects HCW well-being.

**Results:**

Between 48.2 and 59.4% of HCWs agreed or strongly agreed that they: found SFA helpful (48.2%), felt comfortable supporting colleagues (59.4%), would recommend SFA (51.2%), and would continue to use SFA principles (57.2%). Non-White HCWs (particularly Black HCWs), those in assistant/technician positions and those who reported attending a greater number of booster sessions were more likely to agree with positive statements about SFA experiences.

**Conclusions and Relevance:**

Given the continued resurgence of public health emergencies, its lasting effects on HCWs, and related emerging challenges, we expect there to be a continued need for support of patient-facing HCWs.

**Clinical trial registration:**

Clinical Trials.gov Number: NCT04723576 Registered on 01/22/2021 Clinicaltrials.govNCT04723576.

**Supplementary Information:**

The online version contains supplementary material available at 10.1186/s12913-025-13268-6.

## Background

Health care worker (HCW) burnout is a longstanding concern [1–4] in the United States. Causes are multifactorial, including clinical and administrative workloads, and lack of public health system leadership support [5, 6]. During the COVID-19 pandemic [7], HCWs reported mental and physical exhaustion, fear of infection, pain of losing patients and colleagues, and moral distress [8–12]. Even after effective vaccines and evidence-based treatments for COVID-19 became available, HCWs continued to report burnout and experiences of trauma and chronic stress [13]. Contributing factors included staffing shortages [14], workplace violence [12], and repeated surges due to emerging variants [15, 16]. 

During the COVID-19 pandemic, our team conducted a cluster randomized controlled trial (cRCT) to address HCW well-being through implementation and evaluation of Stress First Aid (SFA) [17]. SFA is an evidence-informed intervention that has been utilized in multiple settings prior to the COVID-19 pandemic, including military personnel, emergency first responders, judicial and probation officers, and wildland firefighters [17–23], to build core competencies in identifying and understanding stress reactions to trauma. SFA includes a framework of peer support actions delivered by individuals without formal mental health training. Based on success in these settings for addressing changing, stressful environments, the SFA intervention seeks to mitigate the psychosocial impact of COVID-19 on diverse frontline HCWs through a train-the-trainer training program, customized manuals, and educational materials. A study of SFA saw increases in self-efficacy, resilience and awareness of organizational support and resources post-implementation [24]. Further, they noted a decrease in burnout post-implementation [24]. The intervention was targeted to benefit HCWs facing added stress during the COVID-19 pandemic, including administrative staff, medical assistants, patient support workers, nursing staff, healthcare providers, and social workers of diverse ages, experience levels and racial/ethnic backgrounds.

In addition to understanding the effects of the SFA intervention on HCW outcomes such as psychological distress, it is also vital to investigate the feasibility and acceptability of SFA to ensure it can be utilized in real-world settings [25, 26]. Although prior studies examined HCW experiences with mental well-being interventions [27, 28], the majority were not conducted during a pandemic. Studies conducted during the COVID-19 pandemic in the US were restricted by setting among a limited range of HCWs [29–34]. To understand how best to improve well-being for HCWs across settings in emergent situations, it is crucial to understand the experiences of a large, multi-site diverse sample of HCWs with SFA.

In this paper, we report on a mixed methods evaluation of the acceptability, usability, and experiences of HCWs with SFA across hospitals and Federally Qualified Health Centers (FQHCs) during the COVID-19 pandemic.

## Methods

We present data from a larger study that evaluated the effectiveness of the SFA intervention in a cRCT, “*COV*ID-19 Protection to *E*nsure *R*esilient *H*ealth *C*are *W*orkers (COVER-HCW)”. The effectiveness findings from the trial have been published elsewhere [35, 36]. This paper will outline specifically the perceptions and experiences of the intervention itself for HCW participants.

For these analyses, we include only HCW participants in the SFA (experimental) arm. This study used a mixed methods approach that analyzed: (1) quantitative post-intervention ratings from HCWs who self-reported attending an SFA session and completed both the pre- and post-intervention surveys, and (2) qualitative post-intervention interviews with HCWs. The research plan was approved by the RAND Human Subjects Protection Committee and, for some sites that were unable to cede to RAND’s Human Subjects Protection Committee, their own institutional review board. The study ran from March 2021 – October 2022.

## Study sites

Our team recruited and engaged 28 sites into the study, including eight pairs of hospitals and six pairs of FQHCs. We sought to ensure balance across region and matched pairs on size, type, and COVID-19 burden. Full details on site and participant recruitment are available from the published protocol [36]. 

## Quantitative data

### Survey development

This current quantitative analysis focuses on survey measures related to HCW experiences with SFA designed by the SFA developers [36] to assess implementation outcomes. These measures included the helpfulness of SFA, perceptions of comfort and support at work, and recommendation of SFA for themselves and their colleagues.

### Survey sample

Leaders at all sites provided names, positions, and e-mail addresses of eligible patient-facing HCWs. We reviewed the list for duplication and eligibility. Participants received a $25 Amazon gift card honorarium for survey completion. All HCWs who completed a pre-intervention survey were eligible for the post-intervention survey and received an additional $25 Amazon gift card honorarium for completion.

### Quantitative data analysis

We calculated descriptive statistics of explanatory factors (e.g., age, race/ethnicity, HCW role, time in current facility, time in current role, and number of SFA booster sessions attended) for SFA respondents at hospitals, FQHCs and overall using chi-squared tests between FQHCs and hospitals. We collapsed questions scored on a 5-point Likert Scale (completely agree to completely disagree) to binary response (completely agree and agree vs. neither agree nor disagree, disagree, and completely disagree). We then calculated descriptive statistics of the explanatory factors and the binary evaluation questions including chi-squared tests between agreement and other responses.

## Qualitative interview data

### Interview protocol development

We utilized a semi-structured interview protocol, which incorporated aspects of the Consolidated Framework for Implementation Research (CFIR) [37, 38], to provide additional context on how HCW well-being is impacted by workplace environment. Interviews explored five key domains for HCWs to describe their experiences with SFA, including COVID-19-related challenges, motivation to participate, the importance of well-being, impact of SFA, and patterns of support.

### Interview sample

At the end of the post-intervention survey, HCWs were asked if they would be willing to be contacted for a post-intervention interview. We purposively sampled a subset of all who agreed to be contacted, seeking wide representation with regards to site, gender, age, race/ethnicity and HCW type. HCWs were initially contacted by email with an interview request and were offered a $50 Amazon gift card upon completion. HCWs who did not respond to the initial email were subsequently contacted by phone. We obtained informed consent from all interview participants prior to starting interviews, which lasted approximately 30-minutes, were conducted over phone or videoconference and audio-recorded. Audio recordings were transcribed and reviewed using interview notes to ensure accuracy. We used Dedoose [39] for data management, coding, and analyzing transcripts.

### Qualitative data analysis

We created a codebook for the analysis of the interviews mapped onto the questions from the interview protocols. The qualitative team, comprised of a senior researcher and a team of five trained coders, co-coded three interviews, discussing any points of disagreement. To ensure rigor, transparency, and reliability of code applications, we calculated interrater reliability after 20% of the transcripts were coded with a pooled Cohen’s *Kappa* coefficient [40]. Coding procedures were discussed and refined until the pooled Cohen’s *Kappa* was > 0.80 [40].

Thematic analysis followed Butler-Kisber’s [41] approach of two core stages of analysis. We began with a coarse-grained approach in which we discussed broadly what was revealed, and/or wrote reflective memos to classify emerging themes. The second, fine-grained phase consisted of identifying specific phrases representing themes.

## Results

### Quantitative results

A total of 862 HCWs received the SFA intervention and completed both the pre- and post-intervention surveys (*N* = 245 for the FQHC sample and *N* = 617 for the hospital sample). The overall response rate for the SFA arm (i.e., the percentage of respondents who completed both surveys) was 29%.

In Table 1, we describe the characteristics of HCWs in the SFA arm, distinguishing between those who reported attending SFA (*n* = 367) and those who reported not attending or who did not know if they attended (*n* = 495). We note that HCWs who reported attending were more likely to work at a FQHC, identify as Hispanic/Latino/a, and to be a prescribing provider or administrator/other professional compared with those who did not attend or did not know if they attended. HCWs who did not attend or did not know if they attended were more likely to be White and to be a nurse.

**Table 1:** HCW sample at SFA sites and SFA attendance

% of HCWs (*n*)	Full Sample (*N* = 862)	Attended SFA (*n* = 367)	Did not Attend SFA or Does Not Know (*n* = 495)	*p*-value
**Type of Health Care Facility**				< 0.001
Federally Qualified Health Center (FQHC)	28.4 (245)	46.6 (171)	14.9 (74)	
Hospital	71.6 (617)	53.4 (196)	85.1 (421)	
**Age Group**				0.92
Less than 31 years	25.9 (223)	24.8 (91)	26.7 (132)	
31–50 years	56.4 (486)	58.0 (213)	55.3 (273)	
51 years and over	17.7 (152)	17.2 (63)	18.0 (89)	
**Gender**				0.98
Female	80.7 (696)	80.7 (296)	80.8 (400)	
Non-Female^a^	19.3 (166)	19.3 (71)	19.2 (95)	
**Race/Ethnicity**				< 0.001
Hispanic or Latino/Latina	18.2 (157)	25.9 (95)	12.5 (62)	
Black	15.3 (132)	15.3 (56)	15.4 (76)	
White	53.9 (465)	46.9 (172)	59.2 (293)	
Other^b^	12.5 (108)	12.0 (44)	12.9 (64)	
**Professional Role**				0.035
Clinician^c^	8.9 (77)	10.4 (38)	7.9 (39)	
Nurse	35.4 (305)	29.2 (107)	40.0 (198)	
Assistant or technician	34.8 (300)	36.2 (133)	33.7 (167)	
Administrative or other^d^	20.9 (180)	24.3 (89)	18.4 (91)	
**Years of Employed at the Site**				0.59
≤ 5 years	62.1 (535)	63.9 (234)	60.8 (301)	
**Years in the Profession**				0.40
≤ 5 years	40.4 (348)	42.9 (157)	38.6 (191)	
**# of Booster Sessions Attended**				< 0.001
None	39.6 (177)	29.2 (107)	87.5 (70)	
1–3 sessions	38.3 (171)	43.9 (161)	12.5 (10)	
4–6 sessions	12.8 (57)	15.5 (57)	0 (0)	
7 + sessions	9.4 (42)	11.4 (42)	0 (0)	

^a^Non-Female includes both male and other gender categories due to small sample sizes. For full sample, Other was a sample of *n* = 7

^b^Other was not defined, but with an option to write in

^c^Clinicians included physicians, nurse practitioners, and physician assistants

^d^Other included patient support specialists, behavioral health specialists, substance use counselors, pharmacists, educators, students, and speech therapists

**Fig. 1:**
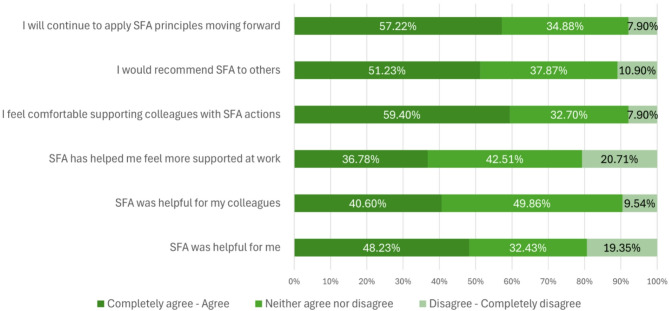
Health Care Worker Experiences with Stress First Aid (SFA) from the Survey

Figure 1 shows that, among those who reported attending SFA training, feasibility and experiences with SFA were reported as mostly positive or neutral as shown in the darkest or darker green portions of the bars. Additionally, more HCWs favored SFA (shown in dark green) than those who did not find it favorable (shown in light green). Of note, over half of HCWs agreed or strongly agreed with statements about feeling more comfortable supporting colleagues (59.4%), recommending SFA (51.2%), and continuing to use SFA principles (57.2%).

In Table 2, we examined HCW agreement with a series of statements related to their experiences with the SFA intervention to determine subpopulation-level trends. Our analyses found significant differences in helpfulness for themselves and for colleagues, feeling supported at work, willingness to recommend, and plans to continue using SFA by race/ethnicity, professional role of the HCW, and the number of booster sessions attended. Non-white HCWs (particularly Black HCWs), those in assistant/technician positions and those who reported attending a greater number of booster sessions were more likely to agree with positive statements about SFA. Those with > 5 years at the organization (56.8%) or in the profession (53.6%) were more likely to agree with the statement “SFA was helpful for me” compared with those with ≤ 5 years at the organization (43.6%) or in the profession (41.4%). There were no other statistically significant differences in responses based on facility type or gender.

**Table 2:** HCW ratings of their experiences with SFA

% of HCWs who Completely Agreed or Agreed (*n*)	Total *N*	SFA was helpful for me	SFA was helpful for my colleagues	SFA has helped me feel more supported at work	I feel comfortable supporting colleagues with SFA actions	I would recommend SFA to others	I will continue to apply SFA principles moving forward
**Overall**	367	48.2 (177)	40.6 (149)	36.8 (135)	59.4 (218)	51.2 (188)	57.2 (210)
**Facility Type**							
FQHC	171	44.4 (76)	39.8 (68)	33.9 (58)	58.5 (100)	49.7 (85)	55.6 (95)
Hospital	196	51.5 (101)	41.3 (81)	39.3 (77)	60.2 (118)	52.6 (103)	58.7 (115)
**Age Group**							
Less than 31 years	91	40.7 (37)	35.2 (32)	35.2 (32)	51.6 (47)	48.4 (44)	56.0 (51)
31–50 years	213	47.9 (102)	42.3 (90)	35.7 (76)	61.0 (130)	48.8 (104)	55.9 (119)
51 years and over	63	60.3 (38)	42.9 (27)	42.9 (27)	65.1 (41)	63.5 (40)	63.5 (40)
**Gender**							
Female	296	50.3 (149)	41.2 (122)	37.2 (110)	60.5 (179)	52.7 (156)	58.8 (174)
Non-Female^a^	71	39.4 (28)	38.0 (27)	35.2 (25)	54.9 (39)	45.1 (32)	50.7 (36)
**Race/Ethnicity**		***	*	*		***	*
Hispanic or Latino/Latina	95	43.2 (41)	42.1 (40)	34.7 (33)	56.8 (54)	50.5 (48)	54.7 (52)
Black	56	69.6 (39)	55.4 (31)	53.6 (30)	71.4 (40)	69.6 (39)	67.9 (38)
White	172	41.3 (71)	33.1 (57)	30.8 (53)	56.4 (97)	41.3 (71)	52.3 (90)
Other^b^	44	59.1 (26)	47.7 (21)	43.2 (19)	61.4 (27)	68.2 (30)	68.2 (30)
**Professional Role** ^**c**^		*	*	**		*	*
Clinician	38	36.8 (14)	36.8 (14)	28.9 (11)	55.3 (21)	42.1 (16)	47.4 (18)
Nurse	107	43.0 (46)	37.4 (40)	34.6 (37)	57.9 (62)	43.9 (47)	53.3 (57)
Assistant or technician	133	57.9 (77)	51.1 (68)	45.9 (61)	66.9 (89)	62.4 (83)	66.9 (89)
Administrative or other	89	44.9 (40)	30.3 (27)	29.2 (26)	51.7 (46)	47.2 (42)	51.7 (46)
**# of Years at Organization**		*					
≤ 5	234	43.6 (102)	38.0 (89)	34.2 (80)	56.4 (132)	47.9 (112)	56.0 (131)
> 5	132	56.8 (75)	45.5 (60)	41.7 (55)	65.2 (86)	57.6 (76)	59.8 (79)
**# of Years in Profession**		*			*		
≤ 5	157	41.4 (65)	35.0 (55)	33.8 (53)	52.2 (82)	46.5 (73)	54.1 (85)
> 5	209	53.6 (112)	45.0 (94)	39.2 (82)	65.1 (136)	55.0 (115)	59.8 (125)
**# of Booster Sessions Attended**		***	***	***	***	***	***
None	107	31.8 (34)	22.4 (24)	19.6 (21)	43.9 (47)	36.4 (39)	42.1 (45)
1–3	161	51.6 (83)	44.7 (72)	37.3 (60)	59.0 (95)	52.8 (85)	58.4 (94)
4–6	57	57.9 (33)	49.1 (28)	49.1 (28)	73.7 (42)	57.9 (33)	68.4 (39)
7+	42	64.3 (27)	59.5 (25)	61.9 (26)	81.0 (34)	73.8 (31)	76.2 (32)

Table Notes: Likert-scale survey responses (completely disagree to completely agree) were collapsed into completely agree/agree and completely disagree/disagree/neither agree or disagree. Asterisk indicates statistical significance (* < 0.05; ** *p* < 0.01; *** *p* < 0.001); p-values are based on chi-square tests demonstrating statistically significant variation related to that entire variable

^a^Non-Female includes both male and other gender categories due to small sample sizes. For full sample, Other was a sample of *n* = 7

^b^Other was not defined, but with an option to write in

^c^Clinicians included physicians, nurse practitioners, and physician assistants. Other included patient support specialists, behavioral health specialists, substance use counselors, pharmacists, educators, students, and speech therapists

### Qualitative results

We conducted 35 interviews with a subset of HCWs who completed the surveys. Table 3 shows that the HCW interview sample is mostly in the middle age group, female, white, and nurses or physicians/NPs/PAs. We focused our qualitative analysis on understanding HCW perceptions of SFA, identifying three themes: (1) SFA provided HCWs with important tools to preserve their well-being; (2) HCWs perceived a positive impact of the SFA intervention on individuals, through increased empathy and support for patients, staff, and peers; (3) HCWs identified positive organizational impacts of the SFA intervention through improved communications and patient care.

**Table 3:** HCW interviewee sample demographics

Characteristic	Total HCWs (*N* = 35)
**Age Group**	
Less than 31 years	7
31–50 years	20
51 years and over	6
Not Reported	2
**Gender**	
Male	8
Female	27
**Race/Ethnicity**	
Hispanic or Latino/a	7
Black	9​
White	12​
Asian	3
Other	4
**Role**	
Clinician^**a**^	10
Nurse/Medical Assistant/Care Manager	17
Behavioral Health	5
Administrative/Other	3

^a^Clinicians included physicians, nurse practitioners, and physician assistants

The first theme described how the SFA intervention nudged HCWs about the importance of addressing stressors at work during the COVID-19 pandemic. For instance, about a quarter of HCWs said the training reminded them to step back and take a break, see Table 4.

**Table 4:** Themes and illustrative quotes for the acceptability, perceived effectiveness, and perceived impact of SFA from HCW perspectives

Subtheme	Quotes
**Theme #1: SFA provided HCWs with important tools**	
Reminder to step back	“I actually do think it’s, it’s a useful thing mainly because sometimes we get just so involved in what’s going on, you’re just wrapped up in it and if you really just like step away have a minute and just kind of regroup yourself to come back.” – Hospital Nurse “Running, running, it feels like you’re running and you’re never stopping yourself for a moment to check in on your inner self, that inner person and just having that be re-informed through the Stress Aid and all the techniques that were said and checking in on yourself and what level, the color system was very helpful….” – FQHC Behavioral Health Specialist
Naming and discussing experiences	“Because you may not even, like I said sometimes you just don’t know the words or have the words or even realize, what you’re feeling is X, Y Z. So, I really would definitely advocate for it, I think it is helpful and beneficial.” –Hospital Behavioral Health Specialist “Most of the time when we were going through these situations, we didn’t have a name for it, but to have a stress training to come in and tell us this is what you’re experiencing. and some of the problems that you’re having at work is due to this. And I think that by identifying it and allowing us a way to plan to attack it in a different situation is always great.” – FQHC Nurse
Demonstrated external support	“I was actually interested in it because I don’t know, it made it seem like somebody out there cared about how we were feeling and what was going on. Somebody would actually listen to us and take how we feel and our stressors into consideration.” - Hospital Patient Care Technician “I remember like I guess I was sort of impressed that [LEADERSHIP] had decided to take that step and like do this thing and kind of like wanting to be more involved in.” – FQHC Psychiatric Nurse Practitioner
**Theme #2: Positive Impact of SFA on Individuals**	
Empathy for patients	“We used it because we went in a different approach to the clients versus how we were doing before. So, we were able to have more understanding and more compassion for the person as far as being like we were able to sympathize more….” – FQHC Nurse “I thought it was nice to kind of bring that kind of problem solving and just like awareness of everyone to the forefront and just to our awareness so we can kind of really assess how we’re treating others around us when we are stressed and things of that nature.” – FQHC Physician
Empathy for staff	“Because you never know what anyone is going through. I mean you can completely hide it, or you can show it completely but like it helped to take a moment and just assess the situation.” – Hospital Nurse “And it just I think it heightens the awareness that because we’re here to help others, right? We’re here to help clients, but it highlighted that, you know, we’re important too, and so it definitely heightened the awareness of looking next to you to see how somebody else’s is doing.” – FQHC Nurse
Supporting fellow peers	“One of the things that I have noticed is when someone is having like a rough day, I’ve definitely seen some of my colleagues like stop and pull it to the side and say, ‘hey, like is everything okay? Is there any way that I can, what can I do to help you?” – Hospital Nurse “I feel like there’s a sort of boundary of everybody’s just doing their thing and if they seem to be really having a hard time, it’s kind of not your business. And so, it seemed like it was kind of a shift of like, hey, if you’re concerned about your colleague, like you should like you should kind of speak up about it.” – FQHC Prescribing Provider “I thought, like we’re all health care professionals and… when the day starts… we just put our heads down and go. I thought… there’s no way that we’re all going to talk about our feelings… I just didn’t think that would happen. And it actually did. People really opened up and it was eye opening.” - FQHC Physician Assistant
**Theme #3: Positive organizational impact of the SFA intervention**	
Communications with peers	“I actually had a colleague come in to talk about something else and she noticed that I wasn’t having a good day and she literally told me like just to stop what I was doing and take three minutes just to stand up and do some breathing with her… I think it heightens the awareness that because we’re here to help others, right? We’re here to help clients, but it highlighted that, you know, we’re important too… it definitely heightened the awareness of looking to see how somebody else’s is doing.” - FQHC Case Manager
Communications across organization	“It allowed the workers to be able to verbalize some concerns to the supervisors and that led to some changes, and I think that communication has improved.” – FQHC Nurse
Improvements in patient care	“The culture that we work in, the more that [SFA’s] the norm and not just some just something that’s being taught, it becomes more useful and more just who we are and not something that someone’s telling us we have to use.” – Hospital Registered Nurse “The quality of care we give to our patients is changing, the way that we address certain situations within our practice has changed, the way that we’ve implemented new types of ways in which to help with psychiatric patients and when they’re having overwhelming or in distress. So, and all of those ways and even our own lives and how we talk to people in and out of work, it’s definitely impacted us.” – FQHC Nurse
**Discussion Quotes**	
Improvements in organizational culture	”I feel like there’s a sort of boundary of like, you know, everybody’s just doing their thing and if they seem to be really having a hard time, like it’s kind of not your business. And so it seemed like it was kind of a shift of like, hey, if you’re concerned about your colleague, like you should like you should kind of speak up about it.” – FQHC Physician “It opened up conversation and I think that that was really important for a couple of our colleagues who needed to take some time off to be able to take care of themselves and you know, it was almost therapeutic in a sense to be able to find that that they’re not alone, you know, to be able to talk to a work colleague that understands and has been through it with them.” – FQHC Medical Assistant

Beyond finding moments of respite, a few HCWs described how SFA allowed them to name their stress and process their experiences. This naming allowed for greater opportunities to relate to others and address stressors. One interviewee described that SFA provided “a way to plan to attack” their stressors, which improved problem solving and stress management, as shown in Table 4. Naming and processing stressful experiences are critical techniques for coping and are useful for well-being and communication [42]. 

Finally, SFA provided a way for HCWs to see pragmatic, external support for their work. Gratitude and the feeling of being heard helped HCWs to continue working in the face of extreme stress. Participants noted that the creation and implementation of a HCW well-being intervention demonstrated care from both researchers and their system leadership, as described in Table 4. A HCW described that their organization leadership’s willingness to implement SFA represented an acknowledgement of and appreciation for their work.

The second theme discussed the impact that individual HCWs felt in their lives and work styles because of the SFA intervention. Many HCWs noted that after the SFA training they were able to demonstrate a greater level of empathy and compassion for their patients. This was described in terms of increased ability to listen, be patient, and understand patient needs, particularly among participants at FQHCs. Several HCWs described a distinct change in their reaction towards patients due to increased empathy and compassion (see Table 4).

This change was also described as improved empathy toward fellow staff. The quotes in Table 4 show that several HCWs noted that they and/or their peers were more likely to intervene when noticing potential peer distress. SFA allowed for more open conversations and realizations of shared experiences, which reinforced peer support dynamics.

Finally, the third theme indicates that, although the SFA intervention was delivered at the individual level, HCWs perceived important organizational impacts as well. First, HCWs noted improved ability and willingness to communicate with one another and to management. In addition, HCWs described how the changes to their communications with patients ultimately improved decision-making and quality of care. HCWs also noted improvements in bidirectional communication and capacity for empathy which work together to form more healthy and supportive work environments, developing a space for SFA to thrive. Improved empathy and communication between staff, leadership and patients was noted to contribute not only to HCW well-being but also to improving the workplace culture of safety and quality, as described in Table 4.

## Discussion

Although COVID-19 has become endemic, public health emergencies will likely continue to arise [43]. Ensuring HCWs’ ability to cope with stressors and burnout will be critical for mitigating the large-scale turnover, workforce shortages and mental health crisis that is still ongoing today. From survey responses, over half of HCWs found SFA training to be useful for their own health and well-being. Most survey respondents noted that they would continue using skills learned from SFA and recommend SFA to others. The demonstrated acceptability of the SFA content is critical for considering SFA as a useful intervention for HCWs. Our qualitative findings show that SFA training provided a common language for HCWs to use to support themselves, each another, and their patients.

While prior effectiveness analyses did not demonstrate an effect overall of SFA on primary outcomes, our secondary analyses showed a potential for SFA to be a useful program for groups that may be at increased risk of stress and burnout. The effectiveness analysis found that younger HCWs from FQHCs saw significant positive effects from SFA [41]. We reviewed results from this subgroup who have completed the SFA training and found no overall age effects for most of the SFA evaluation questions. However, the subgroup analysis found that, among HCWs at FQHCs, those who were older were more likely to report feeling comfortable supporting colleagues with SFA compared with younger HCWs. The support of more experienced colleagues may have contributed in part to our qualitative findings that described interviewees’ perceptions of organizational changes, such as the creation of a more empathetic culture. These changes, when championed and encouraged by leadership (generally comprised of more experienced staff), lead to organizational change [44] and practice diffusion [45, 46]. 

Identifying which types of participants most benefit from SFA is critical. Notably, our findings show that racial/ethnic minoritized HCWs and those in technician or medical assistant roles had more positive experiences with SFA. These groups are disproportionately affected by stress within health care workplace environments [47]. The impact of SFA on these more-vulnerable HCWs, who may experience both higher job strain and lower decision latitude [48], may stem from a greater need for support, making them potentially more open to SFA. Miu and Moore describe the need for additional supports for HCWs of color, particularly the importance of peer support and institutional commitment to well-being, as demonstrated by more positive perceptions of SFA among HCWs of color in this sample [49]. 

The reported usefulness among HCWs from key populations and the positive experiences with SFA reported by a diverse sample of HCWs supports that SFA may be acceptable and feasible for utilization among HCWs in hospitals and FQHCs depending on workplace conditions and program engagement. Given the positive influences of SFA on well-being and potential organizational benefits, it is critical to consider how SFA or similar interventions can be implemented in health care organizations. Importantly, interventions cannot be done in a vacuum. Mercado, et al. found that self-care interventions themselves were not associated with lower stress or burnout rates. However, interventions that improved workplace quality of life, such as peer connectedness and supportive environments, were associated with lower burnout [50]. Therefore, commitment to well-being demonstrated by implementing SFA is best complemented by other workplace improvements for well-being.

### Limitations

Our study took place during the COVID-19 pandemic, a unique period of time with inherent uncertainty, given changes in the pandemic and shifting HCW challenges. Site champions often had to be rapidly deployed to support emergency conditions and therefore, were sometimes unable to conduct or participate in SFA trainings. This resulted in variable adherence to the intervention, with particularly low adherence in hospitals. Thus, SFA was probably not as effective as it could have been. However, our findings are an important contribution because they were conducted in a real-world pandemic setting, reflecting likely adherence and impact if an intervention is needed quickly. Longer term follow-up would be necessary to determine whether interventions such as SFA reduce staff turn-over. Additionally, it is important to note that we restricted our analyses to those who attended SFA based on self-report rather than actual attendance. We selected this approach given that there is no point to understanding subjective views on SFA if the respondent does not remember attending SFA. Next, it is important to note that the qualitative reflections analyzed may be representative primarily of those who felt positively about SFA due to the convenience sample for interviews. These findings are still valuable as the reflections of those who found SFA useful emphasize the beneficial intervention components to be emphasized in future interventions. Finally, because of the convenience nature of the subsample for the qualitative interviews compared to the comprehensive trial survey sample, the demographics for the interviews and surveys respectively may not be fully aligned and representative of one another.

## Conclusions

Given the ongoing effects of the COVID-19 pandemic on HCWs, and related emerging challenges such as worsening workplace violence, we expect there to be a continued need for support of HCWs. While prior effectiveness analyses did not find an overall effect of SFA on primary outcomes [36], the current analyses of secondary outcomes found the intervention to be rated as useful and helpful for a large and particularly vulnerable portion of our sample, particularly racial/ethnic minoritized HCWs and those in service-level positions. The findings of this rigorous, mixed methods evaluation can inform future research of peer support interventions to improve HCW well-being in health care settings, particularly as a pathway for improving health care workplace culture emphasizing collegial support, along with leadership-driven support practices.

## Supplementary Information

Below is the link to the electronic supplementary material.

Supplementary Material 1

Supplementary Material 2

## Data Availability

Raw data used to support the findings of this study are not publicly available to preserve individuals’ and institutions’ privacy.

## Declarations

### Data sharing statement

Data from this trial were deposited into PCORI’s archive in accordance with their data sharing policy.

### Disclaimer

The statements presented in this article are solely the responsibility of the authors and do not necessarily represent the views of the Patient-Centered Outcomes Research Institute (PCORI), its Board of Governors or Methodology Committee.
