# Supplementary material for: Health care worker experiences with a brief peer support and well-being intervention during the COVID-19 pandemic
Source: BMC Health Serv Res. 2025 Sep 30;25:1253. doi: 10.1186/s12913-025-13268-6 (PMC12482828; doi:10.1186/s12913-025-13268-6)
Supplement: Supplementary file 1 — Supplementary Material 1 [file 12913_2025_13268_MOESM1_ESM.docx]

1. **Post-Intervention Health Care Worker Interview Protocol (SFA Sites)**

**Verbal Consent Procedures**

**Purpose:**The purpose of today’s interview is to learn about your experiences with Stress First Aid, COVID-19, system-level perceptions of supportive leadership and organizational culture, as well as role clarity and team cohesion at the unit level.

**Participation is voluntary:**Your participation in this interview is entirely voluntary.  We would like to have your responses to all of the questions; however, you do not have to answer questions about any topics you do not want to discuss.  There are no right or wrong answers.

**Confidentiality:**I want to assure you that your responses to these questions will be strictly confidential. RAND will use the information you provide for research purposes only. We will not disclose your identity to anyone outside our research project staff. Additionally, please do not mention any of your colleagues by name during the interview.

**How we will use the data:**Your participation will help us understand how the well-being of Health Care Workers is impacted by the workplace environment. These interviews will aid in understanding how particular contextual workplace environmental factors could intersect with the implementation and impact of the intervention and its effectiveness.

**Your rights as a participant:**If you have questions about your rights as a research participant or need to report a research-related injury or concern, you can contact RAND's Human Subjects Protection Committee toll-free at (866) 697-5620 or by emailing hspcinfo@ rand.org. If possible, when you contact the Committee, please reference Study: 2020-N0697.

**Audio recording:**As mentioned to you when you were scheduled, we would like to record the interview today to ensure that we capture everything that is said.  We will destroy the recording as soon as we determine that we have captured everything in our notes. Do you have any questions before we begin? If not, do we have your permission to record our conversation?

**Introduction**

1. I’d like to start by asking you about your role and responsibilities at your facility?
   1. How long have you been at your facility?
2. This past year has been a particularly challenging one for health care workers. How have you experienced this past year?
   1. How would you describe your experiences as a health care worker over the course of the pandemic?
   2. How would you describe experiences of being a health care worker in your community over the course of the pandemic?
   3. What kinds of stress have you been experiencing?
   4. How have you been coping with stress?
   5. What is your sense of how your colleagues have been coping with stress?
3. Has your facility provided any additional support for health care workers (e.g., training sessions, guidance on reducing stress, changes to work schedules) prior to implementing Stress First Aid? If so, how helpful was it?
   1. Prior to Stress First Aid, how did you and your colleagues cope with stress on the job? How (well) did you support one another?
   2. Prior to Stress First Aid, how did you and your colleagues cope with stress on the job? How (well) did you support one another?
   3. Had your facility provided any additional support prior to implementing Stress First Aid? If so, how helpful was it?
   4. Did you facility offer vaccination for COVID-19?
4. Please walk me through a typical day during the height of the COVID-19 pandemic.
   1. Were you able to get enough resources and PPE?
   2. How did you and your colleagues reach decisions about patient care?
   3. How did those decisions sit with you?
   4. How did you/were you able to decompress after a day at work?
   5. How else have you been able to cope with stress?
   6. How/has the situation changed over time?
5. What are some barriers to seeking help to support your mental well-being?
   1. Lack of time?
   2. Cost?
   3. Lack of availability of help?
   4. Concerns over credentialing/licensing if you seek help?
   5. Concerns over what others might think (i.e., stigma)?
   6. Concerns that seeking help is a sign of weakness?

**Experiences with Stress First Aid**

1. Let’s think about when you initially heard about Stress First Aid. What were your initial impressions of it?
   1. How would you describe it now in your own words?
   2. What worked well about the training for Stress First Aid?
   3. What was unclear or less effective about the training?
   4. Were you given any protected time to participate in Stress First Aid training?

1. How would you describe the impacts of Stress First Aid?
   1. Have you noticed your colleagues using Stress First Aid training?
   2. How would you describe how Stress First Aid has been woven into your day-to-day tasks?
   3. Can you recall a specific situation where you used the Stress First Aid training? [Please describe in detail]
   4. Thinking about the onset and impacts of that stressful situation (i.e., the stress continuum), were there any points in particular where the training of Stress First Aid helped lessen the stress?
   5. If you noticed an impact, was it more for yourself or for your colleagues?
2. Do you think Stress First Aid has improved your overall health and well-being of your fellow health care workers?
3. Have you noticed any changes to your behavioral health?
4. Mental well-being?
5. Physical well-being?
6. Have you sought any additional care for a health issue [since the implementation of Stress First Aid]?
7. Have you noticed any changes in how other health care workers have been delivering care to patients? In what ways?
8. Patient safety?
9. Patient care experiences?
10. Did you notice if certain types of colleagues responded more positively to Stress First Aid than others/was Stress First Aid more effective for certain colleagues than others?
11. Why do you think this was the case?
12. How do you think Stress First Aid could be made more effective for all health care workers at your facility?

**Sustaining Stress First Aid**

1. Would you advocate for the continuation of SFA?
2. If so, how would you go about advocating for Stress for Aid?
3. What kinds of support would be needed to continue Stress First Aid at your facility?
4. Do you think that Stress First Aid has had an impact on your organization as a whole? If so, how?
5. An increased focus on health care worker well-being?
6. Enhancement of peer support?
7. Changes to workplace morale?
8. More safe discussions of safety and other interpersonal events?
9. When do you think health care workers should be trained for Stress First Aid?
10. What would you change about Stress First Aid?
11. What are your thoughts on follow-up training or refresher courses?
12. Is there anything that would be helpful for coping with the stress of your work?
13. Are there other factors at your facility that you think would help you and your colleagues better manage the stress of your job?
14. Is there anything we did not cover today that you think would be important for us to understand what works well about Stress First Aid and what could be improved?
