# Supplementary material for: Health care worker experiences with a brief peer support and well-being intervention during the COVID-19 pandemic
Source: BMC Health Serv Res. 2025 Sep 30;25:1253. doi: 10.1186/s12913-025-13268-6 (PMC12482828; doi:10.1186/s12913-025-13268-6)
Supplement: Supplementary file 2 — Supplementary Material 2 [file 12913_2025_13268_MOESM2_ESM.docx]

**Draft Survey Instrument: COVER - HCW**

We are interested in understanding healthcare workers’ experiences during the COVID-19 pandemic. The questions below ask about different aspects of your experience and well-being and are meant to be answered in a general sense, taking into account the COVID-19 pandemic, but not specifically focusing on it.

Please tell us about your experiences with COVID-19.

1. *In the last 6 months, about how many patients have you seen with a diagnosis of COVID-19 (confirmed or presumed)?*

⭘ None

⭘ 1-10

⭘ 11-100

⭘ More than 100

1. *Currently at your organization, are you able to obtain COVID-19 testing, even if you are asymptomatic?*

⭘ Yes

⭘ No

⭘ Don’t know

1. *In the past 6 months, how many times have you been tested for COVID-19 at work or elsewhere?*

⭘ 10+ times

⭘ 7-9 times

⭘ 4-6 times

⭘ 2-3 times

⭘ Once

⭘ Never

1. *Have you ever tested positive for COVID-19?*

⭘ No

⭘ Yes – I did not need to be admitted to the hospital

⭘ Yes – I was admitted to the hospital

⭘ N/A – I haven’t been tested

⭘ Prefer not to respond

1. *How worried are you that your work exposure may cause you to get sick from the coronavirus (COVID-19)?*

⭘ Extremely worried

⭘ Very worried

⭘ Somewhat worried

⭘ Not very worried

⭘ Not at all worried

1. *Currently, how worried are you about having appropriate PPE to do your job?*

⭘ Extremely worried

⭘ Very worried

⭘ Somewhat worried

⭘ Not very worried

⭘ Not at all worried

1. *To what extent do you feel that you have adequate support from your organization’s leadership to help you care for patients with COVID-19?*

⭘ Extremely supported

⭘ Very supported

⭘ Somewhat supported

⭘ Not very supported

⭘ Not at all supported

1. *In the past 6 months, how often have you felt that your work makes a significant contribution?*

⭘ Never

⭘ Rarely

⭘ Sometimes

⭘ Usually

⭘ Always

⭘ N/A – I am not directly involved in patient care

1. *In the past 6 months, how often have you felt you could not provide appropriate care to patients with COVID-19 (or those suspected of having COVID-19) due to lack of equipment or other resources?*

⭘ Never

⭘ Rarely

⭘ Sometimes

⭘ Usually

⭘ Always

⭘ N/A – I am not directly involved in patient care

1. *Moral distress is a form of distress that occurs when you believe you know the ethically correct thing to do, but something or someone restricts your ability to pursue the right course of action. Please circle the number (0–10) on the thermometer that best describes how much moral distress you have been experiencing related to work in the past 2 weeks including today.*

*
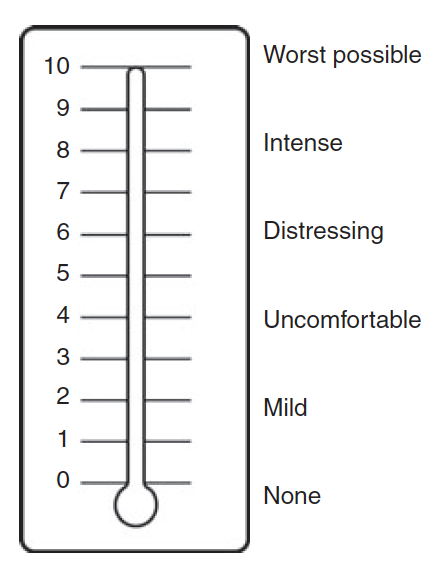
*

1. *Have any of your family members, close friends, and/or colleagues been diagnosed with COVID-19?*

⭘ No

⭘ Yes, one family member, close friend or colleague

⭘ Yes, more than one family member, close friend or colleague

1. *How worried are you that you may expose a family member, close friend or colleague to COVID-19?*

⭘ Extremely worried

⭘ Very worried

⭘ Somewhat worried

⭘ Not very worried

⭘ Not at all worried

1. *How worried are you that your household finances will be impacted negatively by the COVID-19 pandemic?*

⭘ My household finances have already been negatively impacted by COVID-19

⭘ Extremely worried

⭘ Very worried

⭘ Somewhat worried

⭘ Not very worried

⭘ Not at all worried

1. *How worried are you that you or members of your household will experience stigma because of your work in healthcare?*

⭘ Extremely worried

⭘ Very worried

⭘ Somewhat worried

⭘ Not very worried

⭘ Not at all worried-

Now we would like to know about your sleep over the past 7 days. Please respond to each question or statement by marking one response per row.

| *In the past 7 days …* | | **Not at all (1)** | | **A little bit (2)** | | **Somewhat (3)** | | **Quite a bit (4)** | | **Very much (5)** | |
| --- | --- | --- | --- | --- | --- | --- | --- | --- | --- | --- | --- |
| 1. I had a hard time getting things done because I was sleepy | | o | | o | | o | | o | | o | |
| 1. I had problems during the day because of poor sleep | | o | | o | | o | | o | | o | |
| 1. I had a hard time concentrating because of poor sleep | | o | | o | | o | | o | | o | |
| 1. I was sleepy during the daytime | | o | | o | | o | | o | | o | |
| Now we would like to know about your mood over the past seven days. Please respond to each question or statement by marking one response per row.   \| *In the past 7 days …* \| **Never (1)** \| **Rarely (2)** \| **Sometimes (3)** \| **Often (4)** \| **Always (5)** \| \| --- \| --- \| --- \| --- \| --- \| --- \| \| 1. I felt fearful. \| o \| o \| o \| o \| o \| \| 1. I found it hard to focus on anything other than my anxiety. \| o \| o \| o \| o \| o \| \| 1. My worries overwhelmed me. \| o \| o \| o \| o \| o \| \| 1. I felt uneasy. \| o \| o \| o \| o \| o \| \| 1. I felt worthless. \| o \| o \| o \| o \| o \| \| 1. I felt helpless. \| o \| o \| o \| o \| o \| \| 1. I felt depressed. \| o \| o \| o \| o \| o \| \| 1. I felt hopeless. \| o \| o \| o \| o \| o \|   Now, we would like to know about your general responses to changes or hardship. Please respond to each statement by marking one response per row.   \|  \| **Not True at All (0)** \| **Rarely True (1)** \| **Sometimes True (2)** \| **Often True (3)** \| **True Nearly All the Time (4)** \| \| --- \| --- \| --- \| --- \| --- \| --- \| \| 1. I am able to adapt when changes occur. \| o \| o \| o \| o \| o \| \| 1. I tend to bounce back after illness or hardship \| o \| o \| o \| o \| o \|  1. Sometimes things happen to people that are unusually or especially frightening, horrible or traumatic. For example: a serious accident or fire; a physical or sexual assault or abuse; an earthquake or flood; a war; seeing someone be killed or seriously injured; having a loved one die through homicide or suicide.   *Have you ever experienced this kind of event?*  ⭘ Yes  ⭘ No (If no, skip to Q30)   \| In the past month have you … \| **Yes** \| **No** \| \| --- \| --- \| --- \| \| 1. Had nightmares about the event(s) or thought about the event(s) when you did not want to? \| o \| o \| \| 1. Tried hard not to think about the event(s) or went out of your way to avoid situations that reminded you of the event(s)? \| o \| o \| \| 1. Been constantly on guard, watchful, or easily startled? \| o \| o \| \| 1. Felt numb or detached from people, activities, or your surroundings? \| o \| o \| \| 1. Felt guilty or unable to stop blaming yourself or others for the event(s) or any problems the event(s) may have caused? \| o \| o \|   Now we would like to know about the social activities you are able to participate in. Please respond to each question or statement by marking one response per row. | | | | | | | | | | |  |
| *I have trouble doing …* | **Never (5)** | | **Rarely (4)** | | **Sometimes (3)** | | **Usually (2)** | | **Always (1)** | |  |
| 1. My regular leisure activities with others | o | | o | | o | | o | | o | |  |
| 1. The family activities that I want to do | o | | o | | o | | o | | o | |  |
| 1. My usual work activities (including work at home) | o | | o | | o | | o | | o | |  |
| 1. The activities with friends that I want to do | o | | o | | o | | o | | o | |  |

Now we would like to know a little about your physical function. Please respond to each question or statement by marking one response per row.

|  | **Without any difficulty (5)** | **With a little difficulty (4)** | **With some difficulty (3)** | **With much difficulty (2)** | **Unable to do (1)** |
| --- | --- | --- | --- | --- | --- |
| 1. Are you able to do chores such as vacuuming or yard work? | o | o | o | o | o |
| 1. Are you able to go up and down stairs at a normal pace? | o | o | o | o | o |
| 1. Are you able to go for a walk of at least 15 minutes? | o | o | o | o | o |
| 1. Are you able to run errands and shop? | o | o | o | o | o |

Now, we would like to know about your general feelings about work and your organization. Please respond to each statement by marking one response per row.

|  | **Strongly Agree (1)** | **Agree (2)** | **Neither Agree nor Disagree (3)** | **Disagree (4)** | **Strongly Disagree (5)** |
| --- | --- | --- | --- | --- | --- |
| 1. My organization makes me feel valued. | o | o | o | o | o |
| 1. I intend to seek employment outside of my company or organization in the next year. | o | o | o | o | o |

1. *Overall, based on your definition of burnout, how would you rate your level of burnout?*

⭘ I enjoy my work, I have no symptoms of burnout

⭘ Occasionally I am under stress and I don’t always have as much energy as I once did, but I don’t feel burned out

⭘ I am definitely burning out and have one or more symptoms of burnout, such as physical and emotional exhaustion

⭘ The symptoms of burnout that I am experiencing won’t go away. I think about frustration at work a lot

⭘ I feel completely burned out and often wonder if I can go on. I am at a point where I may need some changes or may need to seek some sort of help

These questions will be administered in the PRE-INTERVENTION survey only

*Now please tell us about you.*

1. *What is your age group?*

⭘ under 30

⭘ 31-40

⭘ 41-50

⭘ 51-60

⭘ Over 60

1. *What is your gender?*

⭘ Male

⭘ Female

⭘ Gender-neutral sex designation

1. *Do you consider yourself to be Hispanic, Latino, or of Spanish origin?*

⭘ Yes

⭘ No

1. *What race or races do you consider yourself to be? [Check all that apply]*

🞏 American Indian or Alaska Native

🞏 Black or African American

🞏 Asian

🞏 Native Hawaiian or Pacific Islander

🞏 White

🞏 Decline to state

🞏 Other, what race(s)? Specify:

1. *What is your living situation? (Check all that apply)*

🞏 I live alone

🞏 I live with a roommate(s)

🞏 I live with a partner or spouse

🞏 I live with school aged children (or younger)

🞏 I live with adult children

🞏 I live with aging parents or other aging relative

🞏 I live with a dependent other

🞏 I live with/am a primary caretaker for someone who is at high risk for severe complications from COVID-19 (e.g. elderly, immune compromised, asthma, other underlying medical condition)

1. *Which job title best describes your current role at this hospital, clinic, or health center?*

⭘ Physician (MD, DO)

⭘ Trainee (intern, resident, fellow)

⭘ Physician Assistant

⭘ Nurse Practitioner

⭘ Clinical Nurse

⭘ Pharmacist

⭘ Physical Therapist

⭘ Occupational Therapist

⭘ Respiratory Therapist

⭘ Medical Assistant

⭘ Technician involved in patient care (e.g. pharmacy, phlebotomy, radiology, paramedic/EMT)

⭘ Behavioral Health Specialist (e.g. social worker, clinical psychologist, etc.)

⭘ Care Coordinator/Care Manager

⭘ Administrative (e.g. registration, unit clerk, etc.)

⭘ Other, please specify

1. *In what care setting(s) do you work? [Check all that apply))*

🞏 Emergency Department

🞏 Urgent Care

🞏 Inpatient Floor (med/surg)

🞏 Intensive Care Unit

🞏 Outpatient Clinic

🞏 Other, please specify

1. *How many years have you been employed at this hospital, clinic, or health center?*

⭘ < 1 year

⭘ 1-5 years

⭘ 6-10 years

⭘ 11-15 years

⭘ >15 years

1. *How many years have you been in your profession (at this or other organizations)?*

⭘ < 1 year

⭘ 2-5 years

⭘ 6-10 years

⭘ 11-15 years

⭘ >15 years

[END Pre-Intervention Survey]

THESE QUESTIONS WILL BE IN POST-INTERVENTION SURVEY ONLY

*Below are a few questions to assess your experience with Stress First Aid. Please read each statement carefully and circle the response that indicates how much you agree or disagree with the statement.*

1. Did you receive Stress First Aid training?

⭘ Yes

⭘ No (skip to Q. 75)

⭘ Don’t know

1. How many Stress First Aid booster sessions did you attend?

⭘ none

⭘ 1-3

⭘ 4-6

⭘ 7 or more

1. Stress First Aid works well for people like me.

⭘ Completely disagree

⭘ Disagree

⭘ Neither agree nor disagree

⭘ Agree

⭘ Completely Agree

1. Stress First Aid can be implemented easily in my organization.

⭘ Completely disagree

⭘ Disagree

⭘ Neither agree nor disagree

⭘ Agree

⭘ Completely Agree

1. I feel comfortable using Stress First Aid with my colleagues.

⭘ Completely disagree

⭘ Disagree

⭘ Neither agree nor disagree

⭘ Agree

⭘ Completely Agree

1. I would recommend Stress First Aid to my colleagues.

⭘ Completely disagree

⭘ Disagree

⭘ Neither agree nor disagree

⭘ Agree

⭘ Completely Agree

[END Post-Intervention Survey]
